# Current Therapies and Potential Strategies for Uveal Melanoma

**DOI:** 10.3390/ddc4020014

**Published:** 2025-04-01

**Authors:** Sarah Scoles, Sanjay Ganesh, Kaori H. Yamada

**Affiliations:** 1 Department of Pharmacology & Regenerative Medicine, University of Illinois College of Medicine, Chicago, IL 60612, USA; 2 Department of Ophthalmology & Visual Sciences, University of Illinois College of Medicine, Chicago, IL 60612, USA; 3 University of Illinois Cancer Center, Chicago, IL 60612, USA

**Keywords:** uveal melanoma, metastasis, VEGF

## Abstract

**Background/Objectives:**

Uveal melanoma (UM) is a rare but deadly ocular cancer. This review summarizes the characteristics of uveal melanoma and current therapeutic options for primary uveal melanoma and metastatic uveal melanoma, and introduces recent development of therapeutic strategies in preclinical animal studies and clinical trials.

**Methods:**

The literature search was conducted to identify relevant articles for UM studies. It was performed using PubMed for articles in English until March 2025. Information on clinical trials was also obtained from ClinicalTrial.gov.

**Results:**

Uveal melanoma originates from melanocytes, similar to skin melanoma. However, uveal melanoma has different mutations from skin melanoma. Thus, chemotherapy and immunotherapy, which are effective for skin melanoma, are ineffective for uveal melanoma. Current therapies for UM include radiation therapy, surgical resection, liver-directed therapies, and recently FDA-approved tebentafusp. Although a wide variety of available and newly developed therapies have been tested in clinical trials for UM, tebentafusp is the only FDA-approved therapy for metastatic UM. Given the excessive expression of vascular endothelial growth factor (VEGF) in UM patients with metastatic diseases, anti-VEGF strategies are being tested in clinical trials and pre-clinical animal models.

**Conclusions:**

This review summarizes an overview of current therapies and the development of therapeutic strategies in clinical trials and pre-clinical animal models. Despite successful control of primary tumors, 50% of UM patients still experience metastasis in the liver. Although tebentafusp improves the overall survival (OS) of a certain population of UM patients, robust strategies for preventing UM metastasis represent a critical unmet need. Further investigations of the heterogeneity of UM cells and mechanisms of UM metastasis are needed in the future.

## 1. Introduction

Uveal melanoma (UM) is the most common intraocular malignancy in adults, with the majority of cases originating in the choroid. Despite advances in early detection and treatment, UM has a high rate of metastasis. Approximately 50% of patients with UM develop metastatic disease, which is associated with poor prognosis [1]. Following diagnosis of their UM metastasis, patients have a median survival period of 3.9 months and a three-year survival rate of 4.3% [2].

The liver is the predominant site of UM metastasis, affecting approximately 89% of patients, followed by the lungs (29%) and the bones (17%) [2]. Early detection and effective treatment can mitigate the possibility of metastasis, significantly improving clinical outcomes. Although the mechanism through which UM metastasis occurs is still largely unknown, there are ongoing promising studies. Metastatic UM is highly vascularized with leaky blood vessels [3,4] due to an excess of vascular endothelial growth factor (VEGF) [5–7]. We recently showed that some UM lines rely on VEGF to permeate through the endothelial barrier in order to increase UM cell migration across the endothelium and into the circulatory system [8].

This review aims to provide a comprehensive overview of the current understanding of the biology of UM and possible mechanisms facilitating UM metastasis. Furthermore, this review will discuss emerging strategies to treat and manage UM and UM metastasis.

## 2. Differences Between Skin Melanoma and Uveal Melanoma

Melanoma originates from melanocytes, the pigment-producing cells responsible for melanin synthesis. Melanocytes transfer these melatonin-containing melanosomes to nearby keratinocytes. This process controls skin pigmentation and color and protects against harmful ultraviolet radiation [9]. Skin melanocytes compose the bottom layer of the skin’s epidermis, and ocular melanocytes are located in the conjunctiva and all areas of the uvea, including the iris, ciliary body, and choroid. UM typically arises in these three locations. UM represents 3–5% of all melanomas and 79–81% of ocular melanomas [10]. While both cutaneous (skin) melanoma and UM have the same origin, they differ in their biological behavior, genetic alterations, and clinical presentation, as discussed below.

Although skin and uveal melanoma both originate from melanocytes, they differ through their associated mutations. Skin melanoma primarily has mutations in proteins associated with the mitogen-activated protein kinase (MAPK) pathway [9]. The MAPK is a pathway responsible for cell growth, differentiation, and survival [9]. A majority of skin melanomas have a mutation in the BRAF kinase, which regulates the MAPK pathway [11–14]. *BRAF* mutation is present in 40–60% of skin melanoma patients and is associated with a shorter OS [9]. Other commonly found mutations, such as *NRAS* [15,16] and *KIT* [17] (Table 1), all regulate the MAPK pathway [9].

For skin melanoma with *BRAF* mutation, selective inhibitors of the BRAF kinase can be used, including vemurafenib (Zelboraf, Genentech, South San Francisco, CA, USA), dabrafenib (Tafinlar, Novartis, Basel, Switzerland), and encorafenib (Braftovi, Pfizer, New York, NY, USA). Inhibitors of the downstream MEK kinase are also used, including trametinib (Mekinist, Novartis), cobimetinib (Cotellic, Genentech), and binimetinib (Mektovi, Pfizer). A combination of BRAF inhibitor (dabrafenib) and MEK inhibitor (trametinib) improved OS of skin melanoma patients with *BRAF* mutation, compared to BRAF inhibitor (dabrafenib) alone (NCT01584648, NCT01597908, NCT01584648, NCT01072175) [18–21]. Other combinations (vemurafenib and cobimetinib) also showed that the combined inhibition of BRAF and MEK is better than BRAF inhibition alone (NCT01689519) [22].

KIT inhibitors such as imatinib (Gleevec, Novartis), sunitinib (Sutent, Pfizer), dasatinib (Sprycel, Bristol Myers Squibb, New York, NY, USA), and nilotinib (Tasigna, Novartis) are used for skin melanoma with *KIT* mutation (NCT00470470, NCT01028222, NCT0042515, NCT01099514) [23–27].

In contrast to skin melanoma, UM does not typically have mutations in these genes. UM primarily has mutations in the *GNA11*, *GNAQ*, *BAP1*, *EIF1AX*, and *SF3B1* genes (Table 1). UM is typically initiated by a mutation in *GNA11* [28] and *GNAQ* [29], with greater than 90% of mutations found in *GNA11* and *GNAQ* [9]. Genes *GNA11* and *GNAQ* encode Guanine Nucleotide-Binding Protein Alpha Subunit (GNA, or G protein), which is responsible for the activation signaling between G protein-coupled receptors (GPCR) and downstream effectors. Activation of GNA11 or GNAQ leads to activation of downstream pathways, including the protein kinase C (PKC) pathway [protein lipase C (PLC)-PKC], MAPK pathway (BRAF-MEK1/2-ERK1/2), and phosphatidylinositol 3 kinase (PI3K) pathway (PI3K-Akt-mTOR) [9,30]. However, less than 10% of UM patients responded to the PKC inhibitor (NCT02601378, NCT01801358) [31–33]. Unlike skin melanoma patients, MEK inhibition did not improve the OS of UM patients (NCT01143402, NCT01974752) [34,35]. Potential reasons for resistance to MEK inhibitors in UM patients can be persisting YAP/TAZ signaling [36], overexpression of DDX43-RAS [37], paracrine effects of neuregulin 1 (NRG1) and hepatocyte growth factor (HGF) [38], or monosomy 3 and mutations in *BRCA1 Associated Protein-1* (*BAP1*) [39].

Inactivating somatic mutations in *BAP1* were found in 18–45% of all primary UM and more than 80% of metastasizing UM [40–43]. *BAP1* encodes a nuclear ubiquitin carboxyterminal hydrolase, one of the deubiquitinating enzymes [44]. BAP1 usually functions as a tumor suppressor, and this mutation has been found to correlate strongly with the development of metastatic disease in UM [40,45].

*Eukaryotic translation initiation factor 1A (EIF1AX)* is another mutation found in 14–21% of UM [43], although it is not commonly linked to metastatic disease. *EIF1AX* encodes a eukaryotic translation initiation factor essential for the translation and transfer of tRNA to the small ribosomal unit. This mutation is typically found in nonmetastatic cases of UM [45]. *EIF1AX* mutations are the most prognostically favorable to common UM mutations [46].

Lastly, *splicing factor 3b subunit 1* (*SF3B1*) is another common mutation found in UM tumors. The SF3B1 mutation is associated with a good prognosis if diagnosed early on and a significantly worse prognosis and development of late metastasis in patients five years after diagnosis [45].

In summary, uveal melanoma has mutations that are different from skin melanoma. Thus, commonly used therapies for skin melanoma, such as inhibitions of MEK [34,35], do not work well for UM patients. In addition, unlike skin melanoma, chemotherapy and immune therapy (except recently approved tebentafusp) are not effective for uveal melanoma [47], although the precise mechanisms of how different mutations cause unresponsiveness to chemotherapy and immune therapy are not fully understood.

## 3. Current Therapies for Uveal Melanoma

**Figure 1.**
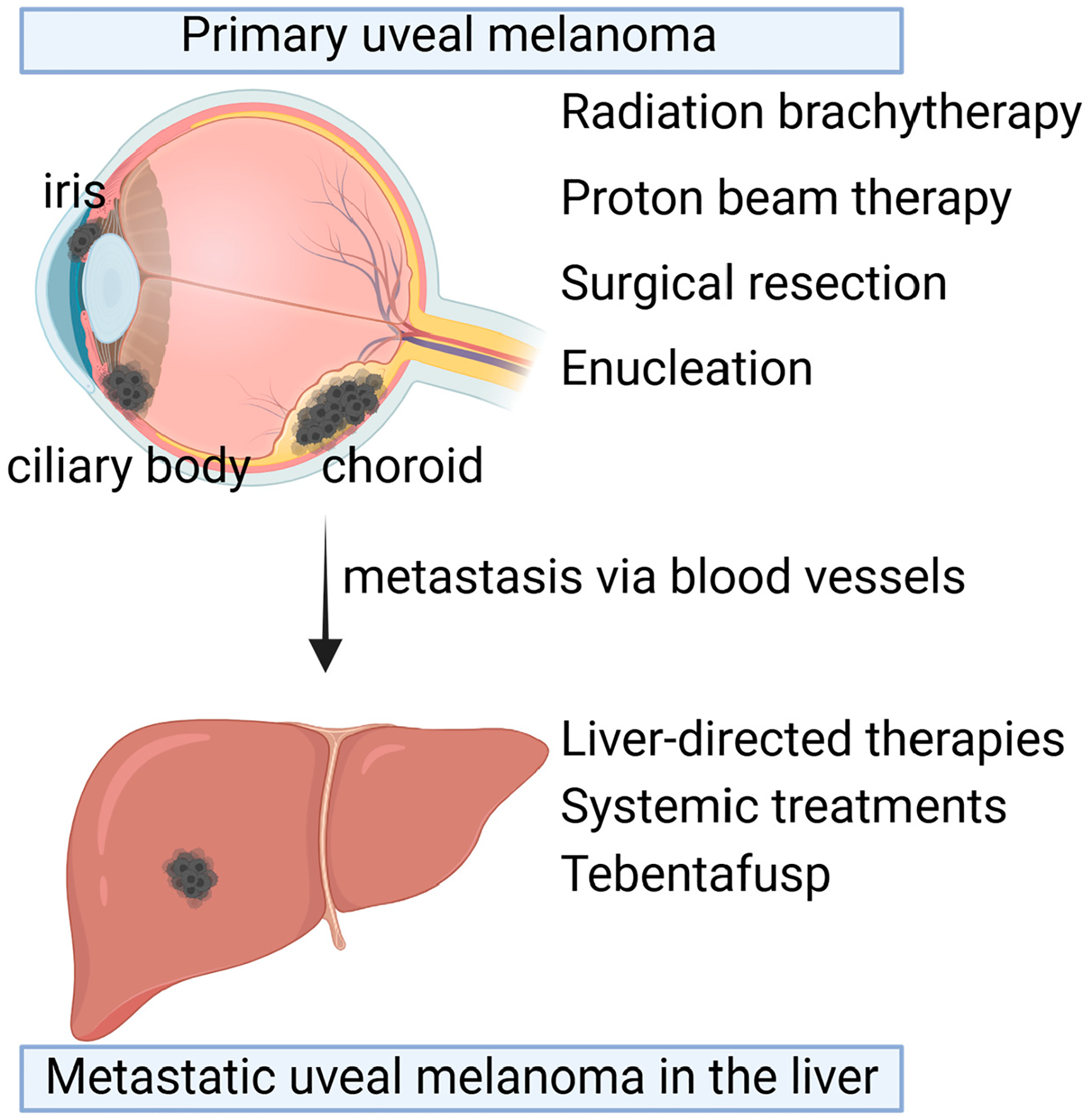
Therapeutic options for primary uveal melanoma and metastatic uveal melanoma. Primary uveal melanoma (UM) occurs in the choroid, ciliary body, and iris in the eyes. Depending on the size and location, primary UM can be treated with radiation brachytherapy, proton beam therapy, surgical resection, or enucleation. Despite the primary tumor treatments, 50% of patients develop metastasis in the liver a long time after primary tumor treatments. Liver-directed therapies or systemic treatments such as Tebentafusp are the available therapeutic options. Created with BioRender.com.

There are various therapies currently approved for the treatment of uveal melanoma tumors, including radiation therapies, surgery, liver-directed therapies, and recently FDA-approved immunotherapy (Figure 1).

### 3.1. Radiation Brachytherapy

Radiation brachytherapy is a commonly used treatment modality for UM [49,50]. This approach involves surgically placing a small radioactive plaque on the sclera adjacent to the tumor, allowing for targeted radiation delivery while minimizing damage to surrounding ocular structures and tissue [1,51]. Plaque brachytherapy has become a standard treatment for localized UM, demonstrating high rates of tumor control and vision preservation [50]. There was no survival difference between patients who underwent enucleation and those treated with iodine-125 plaque brachytherapy [52].

Radiation-induced complications include poor visual outcome, radiation-induced cataracts, vitreous hemorrhage, neovascular glaucoma, secondary glaucoma, retinal detachment, macular edema, and radiation retinopathy [53]. Among them, macular edema and radiation retinopathy, which cause poor visual outcomes, can be dissolved by anti-VEGF therapy. In the randomized Phase IIB trial (NCT0222610), a monthly injection of ranibizumab (anti-VEGF therapy) significantly improved visual outcomes [54]. Corticosteroids, such as dexamethasone (also known as Dexasone, Hexadrol, and Baycadron) and triamcinolone acetonide (Kanalog, Bristol-Myers Squibb), are also used for radiation-induced macular edema and maculopathy [55–60]. Both bevacizumab and corticosteroid injections reduced central foveal thickness and improved some patients’ visual improvements without showing differences or advantages [59]. A combination of bevacizumab and corticosteroid is also beneficial for UM patients in treating severe radiation maculopathy [58].

After plaque radiotherapy, 29 (8.5%) of 43 patients experienced local recurrence [61]. Recurrence occurred with significantly higher frequency when the anterior tumor edge involved the ciliary body [61]. Local recurrence increases the risk of metastasis [62].

### 3.2. Proton Beam Therapy

Proton beam therapy is another form of radiation therapy that is commonly used to treat UM [1,51]. Common indications for the use of proton treatment in UM include small tumors in the posterior pole poorly accessible to plaque treatment, tumors at the posterior pole affecting the fovea, and large anterior tumors traditionally too large for brachytherapy [63]. This treatment option delivers precise, high-energy proton beams to the tumor with the goal of minimizing unwanted radiation exposure to surrounding healthy tissues. While proton beam therapy and plaque brachytherapy are similar, they have key differences with important clinical considerations. Proton beam therapy is more precise and is particularly useful in cases where the tumor is near critical ocular structures, such as the macula and optic nerve, where preserving nearby healthy tissue is vital for maintaining visual function [64]. However, for tumors further from these structures, radiation brachytherapy delivers a dosage closer to the prescribed dose [64]. Additionally, the location of the tumor (temporally vs. nasally) can also impact which radiation treatment option would be more effective.

The downside of this therapeutic option is that most patients become blind after proton therapy [65]. And small populations (1.5%) of patients still experience local recurrence [66,67].

### 3.3. Surgical Resection

Surgical resection of UM tumors may be considered in select cases of UM, particularly when the tumor is small and far from critical ocular structures [68]. This treatment option used to be more common; however, it has been slowly phased out with the advent and success of radiation therapy options [68].

### 3.4. Enucleation

This method was more commonly used prior to plaque brachytherapy and involved the removal of the eye globe—despite the consequences of extremely poor vision and decreased quality of life [1]. Enucleation is still used in cases of very large tumors, where the risk of metastasis is high and it is not worth the time it takes for radiation therapies to take effect [69]. Even after radiation therapies, some patients (7.3~10.3%) experienced local recurrence [62,70,71] and required secondary enucleation [72]. After enucleation, local recurrence in the orbital is rare (<1%); however, it still occurs even without signs of optic nerve invasion or extrascleral extension [73].

### 3.5. Liver-Directed Therapies

Despite the success of primary tumor treatments above, 50% of UM patients still experience metastasis [49]. Given the frequent occurrence of liver metastasis, liver-directed therapies are often used, such as microwave ablation, radio-frequency ablation, and surgical resection [51]. Transarterial chemoembolization (TACE) is another option. During TACE, a combination of chemotherapy drugs and embolic agents that block blood flow is injected directly into the artery supplying blood to the tumor. In addition, selective internal radiotherapy (SIRT), isolated hepatic perfusion (IHP), and percutaneous hepatic perfusion (PHP) are also utilized [51].

A melphalan/hepatic delivery system (HDS) (HEPZATO KIT, Delcath Systems, Inc., New York, NY, USA) provided clinically meaningful response rates and demonstrated a favorable benefit–risk profile in patients with unresectable metastatic UM (NCT02678572) [74]. Melphalan/HDS was approved by the FDA in August 2023 for UM patients with liver metastasis.

### 3.6. Immunotherapy

Recently, the immunotherapy tebentafusp showed an improvement in the OS of UM patients with metastasis (NCT02570308 and NCT03070392) and was approved by the FDA [75–78]. Tebentafusp is a bispecific molecule that targets the gp100 peptide presented by HLA-A*02:01 molecules on tumor cells and engages CD3 on T cells, thus connecting tumor cells with T cells to help the immune system target and destroy melanoma cells [79]. A follow-up study for UM patients who received tebentafusp treatment showed promising survival [80]. One drawback may be skin rashes, as tebentafusp induces T-cell recruitment to skin melanocytes [81]. A recent study further showed that tebentafusp treatment leads to M2-to-M1 macrophage reprogramming, and a combination of tebentafusp with interleukin-2 (IL-2) may enhance benefit in UM patients with high levels of tumor-associated macrophages [82]. Although tebentafusp is very promising, the treatment is limited to patients with HLA-A*02:01. HLA-A*02:01 is a human leukocyte antigen (HLA) class I molecule involved in the presentation of antigenic peptides to CD8+ cytotoxic T lymphocytes. HLA-A*02:01 allele is common in Caucasians (96%) and Native Americans (94%) but less common in Asians [83]. For example, only 47% of Japanese, 23% of Singapore Chinese, and 4% of North Indians have HLA-A*02:01 [83]. For other patients, effective systemic treatment is still needed, as discussed below.

Overall, treatment options for uveal melanoma are greatly dependent on the tumor’s location and size. Patients with very large tumors may have a better prognosis with enucleation, especially if the UM has not metastasized yet. Whereas, if a patient has a tumor closer to the macula and optic nerves, their treatment options are limited due to its sensitive location. In the majority of cases, patients are also given the option of having radiation or enucleation treatment instead of other methods. There are various approved treatment methods for UM, and several novel therapies are being developed and tested.

## 4. Therapies Undergoing Testing in Clinical Trials

In clinical trials, a variety of therapies have been tested for UM, including the aforementioned inhibitions of MEK (NCT01143402, NCT01974752, NCT02768766) [34,35] and PKC (NCT02601378, NCT01801358) [31–33]. In this section, we summarize other therapies for UM in clinical trials. Clinical trials with results in published papers are summarized in Table 2.

### 4.1. Kinase Inhibitors

Tyrosine kinase inhibitors have been tested or are in ongoing tests in clinical trials for UM patients. These tyrosine kinase inhibitors include FDA-approved drugs for other solid cancers, such as crizotinib (Xalkori, Pfizer), sunitinib (Sutent, Pfizer), entrectinib (Rozlytrek, Roche, Basel, Switzerland), cabozantinib (Cabometyx, Exelixis, Alameda, CA, USA), and axitinib (Inlyta, Pfizer) [84]. The use of adjuvant crizotinib did not improve recurrence-free survival (RFS) of patients with high-risk UM (NCT02223819) [85]. However, the other two tyrosine kinase inhibitors show some hope. In a retrospective study comparing high-risk UM patients who received adjuvant sunitinib with institutional controls, the sunitinib group had better OS [86]. A combination of entrectinib with apoptosis inducer PAC-1 was tolerated with no treatment-related grade >3 toxicities, and stable disease was observed in four out of six patients, with a median progression-free survival (PFS) of 3.38 months (95% CI at 1.6–6.5 months) (NCT04589832) [87]. These promising results warrant further clinical investigation of tyrosine kinase inhibitors. Another tyrosine kinase inhibitor, cabozantinib, showed clinical activity in patients with metastatic melanoma, including UM (NCT00940225) [88]. However, in a randomized Phase II trial, cabozantinib did not improve PFS but increased toxicity relative to temozolomide/dacarbazine in metastatic UM (NCT01835145) [89].

Investigational tyrosine kinase inhibitors, including sitravatinib (MGCD516, Mirati Therapeutics, San Diego, CA, USA), cediranib (AZD2171, AstraZeneca, Cambridge, UK), and NN3201 (Novelty Nobility, Seongnam-si, Republic of Korea), are also undergoing testing in ongoing clinical trials.

FDA-approved MEK inhibitors trametinib (Mekinist, Novartis), selumetinib (Koselugo, AstraZeneca), and binimetinib (Mektovi, Pfizer) are also being tested for UM, but no improvements have been observed in PFS [34,35]. Investigational PKC inhibitors darovasertib (IDEAYA Biosciences, South San Francisco, CA, USA) and sotrastaurin (AEB071, Novartis) showed modest clinical activity in metastatic UM [31–33].

An inhibitor for downstream ERK1/ERK2, ulixertinib (BVD-523, Biomed Valley Discoveries, Kansas City, MO, USA), did not show clinical activity in UM patients (NCT03417739) [90]. Focal adhesion kinase (FAK) inhibitors defactinib (Verastem Oncology, Needham, MA, USA), ifebemtinib (IN10018, InxMed, Beijing, China), and PI3K inhibitor roginolisib (IOA0244, iOnctura, Geneva, Switzerland) have been developed and are in ongoing clinical trials for UM.

FDA-approved multi-kinase inhibitor sorafenib (Nexavar, Bayer, Leverkusen, Germany) showed non-progression at 24 weeks in 31.2% of UM patients; however, 41.4% of patients required dose modifications due to toxicity and no improvement in health-related quality of life was shown [91]. Sorafenib was also tested in combination with carboplatin and paclitaxel in metastatic UM, but only minor tumor responses and stable disease were observed in some patients (NCT00329641) [92].

FDA-approved mTOR inhibitor everolimus (Afinitor, Novartis) was tested as a combination with pasireotide, a synthetic somatostatin, in metastatic UM but showed limited clinical benefit (NCT01252251) [93]. The addition of everolimus to carboplatin, paclitaxel, and bevacizumab failed to improve outcomes, with increased toxicity in metastatic melanoma (NCT00976573) [94].

In short, kinase inhibitors have shown varying degrees of efficacy and safety in clinical trials for UM, with some promising results warranting further investigation.

### 4.2. Immunotherapies

In addition to tebentafusp, mentioned above, immunotherapies targeting programmed death receptor-1 (PD-1) and cytotoxic T-lymphocyte-associated protein 4 (CTLA-4) have been tested in clinical trials [95]. Humanized monoclonal antibodies targeting PD-1 include nivolumab (Opdivo, Bristol-Myers Squibb Co.), pembrolizumab (Keytruda, Merck & Co., Inc., Rahway, NJ, USA), durvalumab (Imfinzi, AstraZeneca), and tislelizumab (Tevimbra, BeiGene, Ltd., Beijing, China). These are FDA-approved drugs for other cancers. The combination of nivolumab (anti-PD-1) and ipilimumab (anti-CTLA-4) for metastatic UM showed a modest improvement in OS over historical benchmarks of chemotherapy (NCT02626962, NCT01585194) [96,97]. The combination of nivolumab and ipilimumab was further tested in combination with percutaneous hepatic perfusion with melphalan for safety (NCT04283890) [98]. Neoadjuvant nivolumab and ipilimumab showed higher response rates but substantial toxicity, whereas treatment with nivolumab monotherapy yielded a modest response and low toxicity in metastatic melanoma patients, including for ocular melanoma (NCT02519322) [99].

Pembrolizumab showed clinical benefit in UM patients without liver metastasis or small metastasis volume (NCT02359851) [100]. The combination of pembrolizumab and multi-kinase inhibitor lenvatinib (Lenvima, Eisai Co., Ltd., Tokyo, Japan) showed tolerability and favorable anti-tumor activity in UM (NCT03006887) [101].

Pembrolizumab was also tested as a combination with entinostat (SNDX-275, MS-275, Syndax Pharmaceuticals, Waltham, MA, USA), an inhibitor of histone deacetylase (HDAC) (NCT02697630) [102,103]. The idea behind it is that BAP1 promotes the expression of key developmental genes regulating the switch from pluripotency to differentiation by preventing the deacetylation of histone H3K27 at gene regulatory regions [104,105]. Thus, the inhibition of HCAC activity can rescue the phenotypes associated with BAP1 deficiency by restoring normal expression of genes [104,105]. As BAP1 deficiency in UM is associated with a metastatic phenotype with poor prognosis [40], HCAC inhibitors, such as vorinostat (Zolinza, Merck & Co., Inc.) and entinostat, have been tested as a monotherapy for uveal melanoma (NCT00121225, NCT03022565, NCT01587352, and NCT00020579), although the results have not yet been published. The combination of pembrolizumab and entinostat in patients with metastatic UM showed an objective response rate of 14%, a clinical benefit rate at 18 weeks of 28%, a median PFS of 2.1 months, and a median OS of 13.4 months (NCT02697630) [102].

Durvalumab was tested together with anti-CTLA-4 tremelimumab in combination with tebentafusp (NCT02535078). Tebentafusp with durvalumab demonstrated promising efficacy for metastatic skin melanoma patients [106].

There are more ongoing clinical trials testing investigational anti-PD-1 drugs for metastatic UM. Anti-PD-1 spartalizumab (PDR001, Novartis) is under development for metastatic melanoma [107]. REGN10597 (Regeneron Pharmaceuticals, Tarrytown, NY, USA) is an investigational, PD-1-targeted and receptor-masked IL-2 drug [108]. This drug is designed to enhance the immune response against cancer cells by targeting the PD-1 receptor on T cells while minimizing systemic toxicity [108]. XmAb23104 (Xencor, Pasadena, CA, USA) is an investigational bispecific antibody targeting PD-1 and ICOS (an immune co-stimulatory receptor) [109,110]. XmAb23104 aims to enhance T-cell activation specifically within the tumor microenvironment by simultaneously targeting these receptors, potentially improving anti-tumor responses [109,110]. Another bispecific antibody, XmAb808 (Xencor), targeting the tumor antigen B7-H3 and CD28 co-receptor on T cells [111], is also in an ongoing clinical trial.

Monoclonal antibodies targeting CTLA-4 include ipilimumab (Yervoy, Bristol-Myers Squibb Co.) and tremelimumab (Imjudo, AstraZeneca) [95]. Ipilimumab was tested in combination with nivolumab, as described above [96–99]. Tremelimumab monotherapy for advanced UM showed manageable toxicity but modest PFS and a lack of responses (NCT01034787) [112].

T-cell engaging agents are a class of immunotherapies designed to enhance the ability of the immune system to target and destroy cancer cells. TYRP1-TCB (RO7293583, RG6232, Roche) targets tyrosinase-related protein 1 (TYRP1) on the surface of melanoma and CD3 on T cells, facilitating the interaction between melanoma and T cells [113]. The safety, tolerability, maximum tolerated dose/optimal biological dose, and pharmacokinetics (PK) of TYRP1-TCB were tested for patients with metastatic melanoma (NCT04551352) [114].

Cancer vaccines are also promising strategies for solid tumors, including melanoma [115]. Six melanoma helper peptides (6MHP), MELITAC 12.1, gp100 antigen, MART-1 antigen, tyrosinase peptide, NA17-A antigen, MAGE-12, multi-epitope melanoma peptide vaccine, and tyrosinase DNA vaccine have been tested or are in ongoing clinical trials for metastatic melanoma patients. Skin and uveal melanoma patients received vaccination of 6MHP and successfully developed antibodies against cancer peptides (NCT00089219) [116]. Gp100 antigen [also known as premelanosome protein (PMEL)], MART-1 (also known as Melan-A), and tyrosinase peptide were tested as a combination with ipilimumab (anti-CTLA-4) in melanoma (NCT00032045). Although vaccination (gp100 antigen/MART-1/tyrosinase) failed to induce a measurable response, a higher change in Th-17 inducible cells and higher baseline C-reactive protein (CRP) levels were positively associated with freedom from relapse [117]. A multi-epitope melanoma peptide vaccine with incomplete Freund’s adjuvant induced certain types of immune cells; however, optimized vaccine regimens need to be determined (NCT00705640) [118]. A tyrosinase DNA vaccine (pINGmuTyr, Ichor Medical Systems, San Diego, CA, USA) administered by electroporation in malignant melanoma patients was found to be safe and resulted in Tyr-reactive immune responses (NCT00471133) [119].

Interferon is also used as an immunotherapy as it stimulates the immune system to fight cancer more effectively. However, adjuvant treatment of interferon-α−2b (IFN-α−2b, Merck & Co.) and low-dose dacarbazine (DTIC-Dome, a chemotherapy medication, Bayer) in metastatic UM patients failed to show a significant difference compared to untreated patients (NCT0110528) [120].

Granulocyte-macrophage colony-stimulating factor (GM-CSF, sargramostim, Sanofi, Paris, France) boosts whole white blood cell counts. Immunoembolization with GM-CSF for UM with liver metastasis was safe and showed efficacy in UM patients (NCT00661622) [121]. High doses of immunoembolization with GM-CSF prolonged the survival of UM patients and possibly delayed the progression of extrahepatic metastases [122,123].

Aldesleukin (Proleukin, Iovance Biotherapeutics, San Carlos, CA, USA) is a synthetic version of IL-2, which helps regulate the immune system. The combination of aldesleukin with ipilimumab showed a 17% complete response rate, compared with 7% and 6% with respect to the combination of ipilimumab and gp100 treatment (NCT00058279) [124].

Other immunotherapy drugs are being tested for UM in ongoing clinical trials, including Obinutuzumab (Gazyva, Roche), SEA-CD40 (Seagen Inc., Bothell, WA, USA), tocilizumab (Actemra, Roche), and nelitolimod (SD-101, TriSalus Life Sciences, Westminster, CO, USA). Obinutuzumab is a humanized monoclonal antibody targeting CD20 on the surface of B-lymphocyte [125]. SEA-CD40 is a non-fucosylated, humanized monoclonal antibody targeting CD40, a co-stimulatory receptor on antigen-presenting cells [126]. Tocilizumab is an immunosuppressive, monoclonal antibody targeting interleukin-6 (IL-6) [127]. Nelitolimod is designed to activate Toll-like receptor 9 (TLR9). This activation induces the production of type I interferons, which play a crucial role in the immune response against cancer [128].

In sum, immunotherapies targeting PD-1 and CTLA-4, including combinations of nivolumab and ipilimumab, have shown varying degrees of efficacy and safety in clinical trials for metastatic UM, with ongoing research exploring additional treatments and combinations.

### 4.3. Chemotherapies

Alkylating agents, such as melphalan and fotemustine (LKT Labs, Saint Paul, MN, USA), lomustine (Gleostine, NextSource Biotechnology Miami, FL, USA), carmustine (BiCNU, AVET Lifesciences, Maharashtra, India) (Gliadel, Azurity Pharmaceuticals, Woburn, MA, USA), and cyclophosphamide (Cytoxan, Baxter Healthcare, Deerfield, IL, USA), are widely used chemotherapies for cancers and are being tested for UM patients in ongoing clinical trials. Melphalan/HDS showed efficacy and was approved by the FDA for UM patients with liver metastasis (NCT02678572) [74]. However, another alkylating agent, fotemustine, for intrahepatic treatment for metastatic UM did not improve OS [129].

Paclitaxel (Pfizer) is also a widely used chemotherapy for cancer treatment. Taxoprexin (DHA-paclitaxel, Protarga, King of Prussia, PA, USA), docosahexaenoic acid (DHA)-conjugated paclitaxel, is less toxic and more effective than paclitaxel [130]. Taxoprexin (i.v.) was safe and well-tolerated in metastatic UM patients and showed efficacy, with 32% of patients achieving stable disease (NCT00244816) [131]. Paclitaxel is a microtubule-stabilizing agent, whereas vincristine is a microtubule-destabilizing agent [132]. Marquibo (vincristine sulfate liposome injection) was well tolerated in melanoma patients with impaired liver function (NCT00506142) [133].

In short, alkylating agents and microtubule regulating agents are widely used chemotherapies for various cancers. Although most chemotherapies do not show efficacy for UM patients, there is some hope in the ongoing clinical trials of chemotherapies for UM.

### 4.4. Cell Therapies

Tumor-infiltrating lymphocytes (TIL), autologous T cells, and autologous dendritic cells (DC) are used as cell therapies for cancers, along with autologous TIL therapy for metastatic UM-mediated tumor regression (NCT01814046) [134]. The personalized IKKβ-matured RNA-transfected DC vaccine, which primes T cells and activates natural killer (NK) cells, has been tested for metastatic UM (NCT04335890) [135]. Lifileucel (Amtagvi, Iovance Biotherapeutics), AloCelyvir (Viralgen Vector Core, San Sebastian, Spain), TBio-4101 (Turnstone Biologics, San Diego, CA, USA), BPX-701 (Bellicum Pharmaceuticals, Houston, TX, USA), and ACTengine (Immatics, Stafford, TX, USA) are in ongoing clinical trials for metastatic UM [136]. Although single intravenous administration of oncolytic adenovirus ICOVIR-5 failed to induce tumor regressions in skin and uveal melanoma patients [137], ICOVIR-5 was further used to modify allogenic bone marrow-derived mesenchymal stem cells and as a cell therapy, in the form of AloCelyvir, in ongoing clinical trials [138].

### 4.5. Viral Therapies

Oncolytic virus therapy uses viruses that selectively replicate within cancer cells, causing them to burst and die. This process also helps stimulate the immune system to recognize and attach to the cancer. Coxsackievirus A21 (CVA21) (Cavatak, Merck & Co.) is an oncolytic virus therapy, targeting intracellular adhesion molecule 1 (ICAM-1) and decay-accelerating factor (DAF), which are abundant on the surface of cancer cells [139]. CVA21 was tested in combination with ipilimumab in UM patients (NCT03408587), but a meaningful clinical benefit was not observed [140]. Other viral therapies tested in clinical trials for UM include RP2 (Replimune, Woburn, MA, USA) and ADV/HSV-tk (Candel Therapeutics, Needham, MA, USA). ADV/HSV-tk uses a replication-deficient adenovirus vector to deliver the herpes simplex virus thymidine kinase (HSV-tk) gene into cancer cells. Once inside the cells, the HSV-tk gene makes the cells sensitive to antiviral drugs like ganciclovir or valacyclovir, which kill the cancer cells [141].

### 4.6. Targeted Cancer Therapy

BAP1 mutation in UM causes dysfunction in the DNA damage response [142]. The enzyme poly ADP-ribose polymerase (PARP) helps repair damaged DNA in the cells; thus, inhibition of PARP prevents cancer cells from repairing their DNA, leading to cancer cell death [143]. PARP inhibitors include olaparib (Lynparza, AstraZeneca) and niraparib (Zejula, GlaxoSmithKline, London, UK). However, niraparib treatment for cancer patients with mutant BAP1 failed to meet the prespecified efficacy end point for response (NCT03207347) [144].

ADI-PEG20 (pegargiminase) works by depleting arginine, an amino acid essential for the growth and proliferation of cancer cells, and has been tested in combination with pemetrexed (antimetabolites) and cisplatin chemotherapy for argininosuccinate synthetase (ASS1)-deficient metastatic UM (NCT02029690). Seven out of ten patients had stable disease with a median PFS of 3.0 months and a median OS of 11.5 months [145].

Targeted delivery of drugs using the cell surface proteins of melanoma has been used to develop novel therapies for UM. DYP688 (Novartis Pharmaceuticals) is an antibody–drug conjugate targeting gp100 [146]. 225Ac-MTI-201 (Modulation Therapeutics, Tampa, FL, USA) targets the melanocortin-1 receptor (MC1R) and uses the alpha-emitting radionuclide actinium-225 [147]. VMT01 and VMT02 (Perspective Therapeutics, Richland, WA, USA) are also targeted radiation for MC1R [148].

Belzupacap Sarotalocan (AU-011, Aura Biosciences, Boston, MA, USA) is a nanoparticle conjugate that selectively binds to cancer cells in the eyes and is activated by light [149,150]. Glembatumumab vedotin (CDX-011, CR011-vcMMAE) is an antibody–drug conjugate targeting GPNMB [151]. Among 35 metastatic UM patients who received glembatumumab vedotin treatment, two patients had confirmed partial responses and 18 had stable disease as the best objective response (NCT02363283) [151].

Alrizomadlin (APG-115, Ascentage Pharma, Suzhou, China) is an orally administrated, selective, small molecular inhibitor of the MDM2 protein, designed to reactivate the p53 tumor suppressor pathway [152], and is in an ongoing clinical trial for skin and uveal melanoma.

Melatonin is an indolamine hormone that has improved survival in previous trials with patients with various cancers and is in ongoing clinical trials for UM patients (NCT05502900) [153].

In summary, various FDA-approved therapies for other cancers or investigational drugs have been tested for UM patients in clinical trials. Although some trials showed moderate efficacy that warrants further investigations, tebentafusp is the only FDA-approved therapy for metastatic UM.

## 5. Metastasis of Uveal Melanoma

Primary UM has a high rate of metastasis, with approximately 32% of cases metastasizing by 5 years, 50% by 15 years, 56% by 25 years, and 62% by 35 years [45]. Despite this, the mechanism by which UM escapes the eye remains largely an area of active study.

In addition to the mutations described above in Section 2, upregulated expressions of genes in UM have been studied [158–162]. High expression of *preferentially expressed antigen of melanoma (PRAME)* in UM is associated with poor outcomes and correlated with extraocular extension and chromosome 8q alterations [158,163]. The first identified differentially expressed genes (DEGs) in high-risk UM compared to low-risk UM are *CDH1* (up), *ECM1* (up), *HTR2B* (up), *RAB31* (up), *EIF1B* (down), *FXR1* (down), *ID2* (down), *LMCD1* (down), *LTA4H* (down), *MTUS1* (down), *ROBO1* (down), and *SATB1* (down) [164,165]. Similar approaches identified different sets of genes; for example, three upregulated genes, *HTR2B*, *AHNAK2*, and *CALHM2*, and six downregulated genes, *SLC25A38*, *EDNRB*, *TLR1*, *RNF43*, *IL12RB2*, and *MEGF10* [166]. Proteomics analysis of UM further suggests upregulated proteins in metastatic UM [167]. These upregulated expressions can be prognostic biomarker and therapeutic targets; however, further investigations are needed to develop therapeutic approaches.

**Figure 2.**
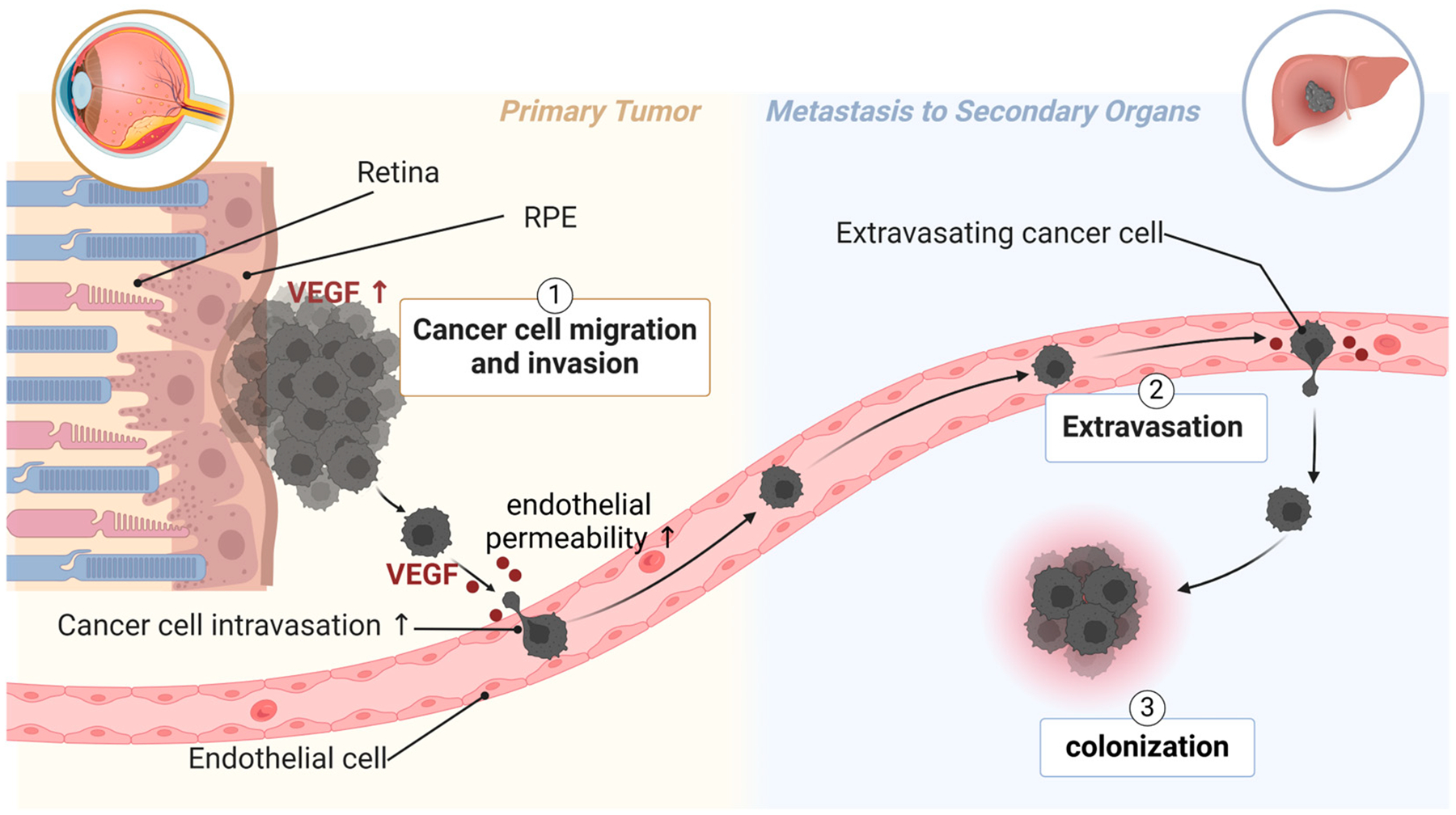
The role of VEGF in UM metastasis. Highly metastatic UM has excessive expression of VEGF in primary tumors. UM secretes VEGF to induce endothelial permeability, thus facilitating intravasation of UM cells into circulation. UM patients with metastasis also have higher levels of VEGF in circulation, which can be from circulating UM cells. Clinical trial studies show that systemic treatment with aflibercept is beneficial for metastatic UM patients. Created with BioRender.com.

UM metastasizes primarily through the bloodstream, as lymphatic vessels do not exist in the eyes [168]. Poor patient survival is associated with high microvascular density in primary tumors from UM patients [3,4]. UM secretes many factors that facilitate endothelial permeability, the adhesion of cancer cells to endothelium, and the digestion of extracellular matrix [53,169,170]. Such factors include VEGF (Figure 2) [167]. UM patients with higher VEGF-A levels (in the aqueous humor [5] or serum [6,7]) and more activated VEGF receptor 2 (VEGFR2) in primary UM tissue [171] have a higher risk of metastasis and poorer survival. VEGF is known to induce endothelial permeability, facilitating the transmigration of UM cells across the endothelium and into distant organs. This underscores the importance of targeting angiogenic factors to limit the spread of UM and improve clinical outcomes.

In clinical trials, systemic treatment of an anti-VEGF drug (aflibercept) showed beneficial effects (e.g., PFS) in patients with stage III and stage IV melanoma of cutaneous or uveal origin NCT00450255) [156]. In a randomized Phase II study, a combination of systemic aflibercept treatment and IL-2 improved PFS compared with IL-2 alone for patients with metastatic UM [157]. In an animal model of orthotopically implanted UM, systemic (i.p.) treatment of anti-VEGF (bevacizumab) inhibited tumor growth, tumor angiogenesis, and metastasis [172]. However, intravitreal injection (i.v.t.) of bevacizumab promoted primary tumor growth in mice [173] and humans [174], which is unexpected and paradoxical. The reasons why bevacizumab (i.v.t.) is ineffective remain unclear. Nonetheless, systemic treatment with anti-VEGF drugs seems to be effective for UM patients with metastasis [156,157], and combinations with stereotactic body radiation therapy (NCT03712904), Nab-Paclitaxel (Abraxane, Bristol-Myers Squibb) (NCT02158520), and cemiplimab (Libtayo, anti-PD-1, Regeneron Pharmaceuticals) (NCT06121180) are in ongoing clinical trials.

In addition to the secreted factors, tumor-derived extracellular vesicles (TEVs) are known to bridge the communication between tumor cells and their microenvironment. UM TEVs contain proteins involved in several cell signaling pathways, including VEGF. UM-derived EVs have been shown to upregulate the expression of VEGF and contribute to increased vascularization through capillary-like networks in endothelial cells [53]. UM-derived EVs increased cell proliferation, migration, invasion, angiogenesis, and metastases compared to EVs from normal choroidal melanocytes [175]. As tumor cells escape into the circulatory system, these TEVs can transfer their material to neighboring cells, which favors tumorigenesis. In addition to UM dissemination, TEV use has been proposed for early detection of UM [53]. TEVs contain various biomarkers in other cancers and other types of clinical information [175]. There is hope that a non-invasive method of early diagnosis can be developed using EVs, which would significantly improve the prognosis of patients if detected earlier in the cancer’s progression.

## 6. Potential Therapies to Prevent Uveal Melanoma Metastases

In preclinical animal models, more strategies to prevent UM metastasis have been tested. In a study by Nhàn et al., we determined that UM cells secrete VEGF to induce endothelial permeability, which facilitated UM cell transmigration across the endothelium [8]. Among the UM cell lines tested, transendothelial migration of MP41 (*GNA11*^*Q209L*^) and 92.1 (*GNAQ*^*Q209L*^ with *EIF1AX*^*G6D*^) were inhibited by anti-VEGF treatment, whereas Mel202 (*GNAQ*^*Q209L/R210K*^, *CDKN2A*^*L65R*^, *SF3B1*^*R625G*^) was not inhibited, suggesting reliance on other pathways. Previously, we developed a novel peptide, KAI, that inhibits the trafficking of the receptor for VEGF, VEGFR2, to the cell surface, thereby blocking VEGF/VEGFR2 signaling [176,177]. Systemic treatment with KAI was found to effectively inhibit tumor angiogenesis and extravasation of skin melanoma in mice [177]. In the animal model of orthotopically injected UM, treatment of tumor-bearing mice with daily eyedrops of KAI reduced the number of mice with circulating tumor cells (CTCs) compared to the no-treatment group, indicating inhibition of UM cells escaping from the eyes by KAI treatment [8]. As the intravasation of UM cells into circulation is an initial step of metastasis, its inhibition can be one of the effective strategies for inhibiting metastasis in distal organs. Other effective strategies include the inhibition of the transition of dormant cancer cells into colonization and the inhibition of cancer growth in the distal organs.

## 7. Methods

We used PubMed to search for the literature describing “uveal melanoma” and “therapy”. The search provided us with 3738 results from 1956 to March 2025. Among them, we read the literature written in English and cited the relevant literature in this review. For recent clinical trials which have not been published yet, we obtained information from ClinicalTrials.gov by searching “uveal melanoma” as a condition/disease and retrieved 238 trials.

## 8. Conclusions

Current therapies for UM include the treatment of primary tumors, mainly by radiotherapy and surgical resection. Despite successful control of primary tumors, 50% of UM patients still experience metastasis in the liver. Once metastasized, liver-directed therapies and/or recently FDA-approved tebentafusp can be employed, which is beneficial for a certain population of UM patients. However, robust strategies for preventing UM metastasis represent a critical unmet need. Although recent clinical trials and preclinical studies showed potential for targeting the VEGF pathway, further investigation of heterogeneity of UM cells with different mutations and different responses to anti-VEGF therapy is needed in the future.

## Figures and Tables

**Table 1.** Mutations in skin melanoma and uveal melanoma.

Mutations Found in Skin Melanoma	Functions	References
*BRAF*	B-Raf kinase regulating MAPK pathway	[11–14]
*NRAS*	Small GTPase	[15,16]
*KIT*	Receptor tyrosine kinase	[17]
Mutations Found in Uveal Melanoma	Functions	References
*GNA11*	The alpha subunit of Guanine nucleotide-binding proteins	[28]
*GNAQ*	The alpha subunit of Guanine nucleotide-binding proteins	[29]
*BAP1*	Deubiquitinating enzyme	[40]
*EIF1AX*	Eukaryotic translation initiation	[48]
*SF3B1*	Essential for pre-mRNA splicing	[48]

**Table 2.** Clinical trials for UM with results.

Drugs	Mode of Action	Phase	References
Darovasertib (LXS196, IDE196)	PKC inhibitor	Phase I (NCT02601378)	[31]
Sotrastaurin (AEB071)	PKC inhibitor	Phase I/II (NCT01801358)	[33]
Selumetinib (Koselugo)	MEK1/2 inhibitor	Phase II (NCT01143402, NCT02768766), Phase III (NCT01974752)	[34,35]
Ranibizumab	Anti-VEGF	Phase II (NCT02222610)	[54]
Crizotinib (Zalkori)	Tyrosine kinase inhibitor	Phase II (NCT0222819)	[85]
Entrectinib	Tyrosine kinase inhibitor	Phase I/II (NCT04589832)	[87]
Cabozantinib	Tyrosine kinase inhibitor	Phase II (NCT01835145, NCT00940225)	[88,154]
Ulixertinib (BVD-523)	ERK inhibitor	Phase II (NCT03417739)	[90]
Sorafenib (Nexavar)	Multi-kinase inhibitor	Phase II (NCT00329642)	[92]
Everolimus (Afinitor, Zortress, Votubia)	mTOR inhibitor	Phase II (NCT01252251, NCT00976573)	[93,94]
Nivolumab (Opdivo)	Anti-PD-1	Phase II (NCT02626962), Phase I/II (NCT04283890), Phase II (NCT02519322, NCT01585194)	[96–99]
Pembrolizumab (Keytruda)	Anti-PD-1	Phase I (NCT03006887), Phase II (NCT02359851, NCT02697630)	[100–102]
Tremelimumab (Imjudo)	Anti-CTLA-4	Phase II (NCT01034787)	[112]
RO7293583	TYRP-1 targeting CD3 T cell engager	Phase I (NCT04551352)	[114]
6MHP	Peptide vaccine	Phase I/II (NCT00089219)	[116]
Gp100 antigen	Peptide vaccine	Phase II (NCT00032045, NCT00084656)	[117,155]
Multi-epitope melanoma peptide vaccine	Peptide vaccine	Phase I (NCT00705640)	[118]
Tyrosinase DNA vaccine	Vaccine	Phase I (NCT00471133)	[119]
Interferon	Interferon	Phase II (NCT01100528)	[120]
GM-CSF	Growth factor	Phase II (NCT00661622)	[121]
Aldesleukin (Proleukin)	A synthetic IL-2	Phase I/II (NCT00058279)	[117]
Melphalan (Alkeran, Evomela)	Alkylating agent	Phase III (NCT02678572)	[74]
Fotemustine	Alkylating agent	Phase III (NCT00110123)	[129]
Taxoprexin (DHA-paclitaxel)	Chemotherapy	Phase II (NCT00244816)	[131]
Marqibo	Vincristine sulfate liposome injection	Phase II (NCT00506142)	[133]
Tumor-infiltrating lymphocytes	Cell therapy	Phase II (NCT01814046)	[134]
Autologous dendritic cells	Cell therapy	Phase I (NCT04335890)	[135]
CVA21 (Cavatak)	Oncolytic virus targeting ICAM1 and decay-accelerating factor (DAF)	Phase I (NCT03408687)	[140]
Niraparib (Zejula)	PARP inhibitor	Phase II (NCT03207347)	[144]
ADE-PEG20	Depleting arginine	Phase I (NCT02029690)	[145]
Glembatumumab vedotin (CDX-011, CR011-vcMMAE)	Antibody-drug conjugate targeting GPNMB	Phase II (NCT02363283)	[151]
Melatonin	Hormone	Phase III (NCT05502900)	[153]
Aflibercept	Anti-VEGF	Phase II (NCT00450255)	[156,157]

## Abbreviations

The following abbreviations are used in this manuscript:

6MHP: 6 melanoma helper peptides
ASS: Argininosuccinate synthetase
BAP1: BRCA1 Associated Protein-1
CRP: C-reactive protein
CTC: Circulating tumor cells
CTLA-4: Cytotoxic T-lymphocyte-associated protein 4
CVA21: Coxsackievirus A21
DAF: Decay-accelerating factor
DC: Dendritic cells
EIF1AX: Eukaryotic translation initiation factor 1A
FAK: Focal adhesion kinase
GM-CSF: Granulocyte-macrophage colony-stimulating factor
GNA: Guanine Nucleotide-Binding Protein Alpha Subunit
GPCR: G protein-coupled receptors
HDAC: Histone deacetylase
HDS: Hepatic delivery system
HLA: Human leukocyte antigen
HSV-tk: Herpes simplex virus thymidine kinase
IFN: Interferon
IHP: Isolated hepatic perfusion
IL: Interleukin
i.v.t: Intravitreal injection
MAPK: Mitogen-activated protein kinase
MC1R: Melanocortin-1 receptor
NK: Natural killer
OS: Overall survival
PARP: Poly ADP-ribose polymerase
PD-1: Programmed death receptor-1
PFS: Progression-free survival
PHP: Percutaneous hepatic perfusion
PK: Pharmacokinetics
PMEL: Premelanosome protein
RFS: Recurrence-free survival
SF3B1: Splicing factor 3b subunit 1
SIRT: Selective internal radiotherapy
TACE: Transarterial chemoembolization
TEV: Tumor-derived extracellular vesicle
TIL: Tumor-infiltrating lymphocytes
TLR: Toll-like receptor
TYRP1: Tyrosinase-related protein 1
UM: Uveal melanoma
VEGF: Vascular endothelial growth factor
